# Lifestyle correlates of dietary patterns among young adults: evidence from an Australian birth cohort

**DOI:** 10.1017/S1368980021003864

**Published:** 2022-08

**Authors:** Tolassa W Ushula, Petra H Lahmann, Abdullah Mamun, William YS Wang, Gail M Williams, Jake M Najman

**Affiliations:** 1 School of Public Health, Faculty of Medicine, The University of Queensland, Brisbane, Australia; 2 Nutrition and Dietetics Department, Faculty of Public Health, Jimma University, Jimma, Ethiopia; 3 Institute for Social Science Research, The University of Queensland, Brisbane, Australia; 4 The Australian Research Council (ARC) Centre of Excellence for Children and Families over the Life Course, The University of Queensland, Brisbane, Australia; 5 The Queensland Alliance for Environmental Health Sciences, The University of Queensland, Brisbane, Australia; 6 Faculty of Medicine, The University of Queensland, Princess Alexandra Hospital, Brisbane, Australia

**Keywords:** Lifestyle correlates, Food Frequency Questionnaire, Dietary patterns, Principal component analysis, Young adults, Australia

## Abstract

**Objective:**

Previous studies of sociodemographic and lifestyle correlates of dietary patterns among young adults have primarily focused on physical activity and smoking, with inconclusive results. This study aims to examine the associations between a broader range of lifestyles of young adults and their patterns of food consumption.

**Design:**

Cross-sectional.

**Setting:**

Brisbane, Australia.

**Participants:**

The data set are from a long running birth cohort study which commenced in 1981. Details of dietary intake and sociodemographic and lifestyle factors were from the 21-year follow-up of the Mater-University of Queensland Study of Pregnancy (MUSP) birth cohort. The effective cohort (*n* 2665, 57 % women) is of young adult offspring. Usual dietary intake was assessed using a Food Frequency Questionnaire (FFQ). Data on sociodemographic and lifestyle variables were obtained from self-reports.

**Results:**

Western and prudent dietary patterns were identified for the combined cohort of women and men using principal components analysis. Multivariable linear regression models were used to examine the associations between lifestyle variables and dietary patterns adjusting for potential confounders. Results from multivariable adjusted models showed that physical activity, watching TV and smoking were strongly associated with each dietary pattern; alcohol consumption and BMI showed weaker associations (*P* < 0·05 for all).

**Conclusions:**

Our study describes a clustering of unhealthy lifestyles in young adults. Young adults with unhealthy lifestyles less often adhere to a healthy prudent dietary pattern and more often an unhealthy Western pattern. Dietary preferences are enmeshed in a lifestyle matrix which includes physical activity, sedentary activity, smoking and alcohol consumption of young adults.

Dietary and other modifiable lifestyle habits including cigarette smoking, alcohol consumption, physical inactivity, snacking, skipping breakfast and higher body weight have been causally linked to long-term adverse health outcomes including morbidity and mortality^(1)^. There is little known about the extent to which dietary patterns are a more general component of lifestyle during young adulthood. Young adulthood is characterised by extensive lifestyle changes and explorations^(2)^. While the underlying contributing factors to the development and clustering of unhealthy lifestyles are the subject of a body of research, socio-cultural and environmental factors are believed to play important roles in these processes^(3,4)^. Unhealthy lifestyles including poor dietary habits, smoking, alcohol consumption and physical inactivity as well as screen-based/sedentary activity can be observed during young adulthood^(5–8)^. These lifestyles, particularly poor dietary habits, sedentary activity and smoking, develop early in the life course and are likely to track through adolescence and into young adulthood^(9–12)^. The multifaceted lifestyles observed during young adulthood may be interrelated and may be associated with poor food choices^(13–17)^ and bad eating habits: skipping meals; eating away from home; and include a reliance on fast foods and energy dense snacks^(6,7)^. It is plausible that dietary habit is part of a broader aspect of lifestyle and that lifestyle interventions may be necessary to facilitate better food choices and better eating habits. In this study, we report findings from the Mater University of Queensland Study of Pregnancy (MUSP) birth cohort to examine the associations between the dietary patterns of respondents and four key lifestyle factors, specifically smoking, alcohol consumption, physical activity and screen-based/sedentary activity in young adult offspring at the 21-year follow-up.

A few studies have specifically focused on lifestyle behaviours and their associations with dietary patterns among young adults^(18)^. There are some studies which examine sociodemographic and lifestyle variables influencing dietary pattern of young adults. We identified seven such studies conducted in the USA, Brazil, Denmark, UK and Canada including men and women aged between 18 and 39 years^(19–25)^. These studies have largely focused on smoking^(18–20,23)^ and physical activity^(19,23,24)^ as lifestyle variables, with conflicting results. For example, in some studies less healthy eating patterns have been associated with lower level of physical activity^(19,23)^ and smoking^(19,20,23)^ in young adults. A high level of physical activity has been associated with increases in prudent dietary pattern scores over time among young women in the UK^(24)^. By contrast, smoking has shown no association with any of the identified dietary patterns in this later study of young women in the UK^(24)^ and in another study of men and women in the USA^(18)^. A few studies that have considered alcohol consumption^(19)^ and sedentary activity^(18,24)^ have not observed association with the dietary patterns they examined.

There is also limited evidence regarding BMI as a possible lifestyle correlate of dietary patterns, particularly during the period of emerging adulthood when weight gain is likely high^(26,27)^. While BMI appears unrelated to changes in dietary patterns over 2 years among young women in the UK^(24)^, other studies that have adjusted for a confounding effect of BMI in predicting dietary patterns have not reported estimates for BMI^(20,22,23)^.

In general, previous studies have focused largely on physical activity and smoking and overlooked other lifestyle factors such as alcohol consumption, sedentary activity and body weight in relation to patterns of eating among young adults. Moreover, there is a mounting body of evidence showing sociodemographic characteristics including older age, female gender and both higher income and education are positively associated with the healthier food choices^(28,29)^ as well as healthier dietary patterns^(20–23)^ in young adults. We hypothesise that both lifestyles and dietary patterns are, in part, a consequence of the social contexts in which a person is reared. In this study, we examine whether dietary patterns are correlated with a range of lifestyles in young adults adjusting for potentially confounding effects of such social contexts including levels of income and education. Specifically, we examine physical activity, sedentary activity, cigarette smoking, alcohol consumption and BMI and their associations with dietary patterns in young Australian women and men at a mean age of 21 years using a large population-based data set.

## Methods

### Participants and design

The present study uses offspring data from the MUSP, a prospective birth cohort study of mother-offspring pairs in Brisbane, Australia. Between 1981 and 1983, all women (*n* 8556) who presented for their first antenatal visit at the Mater Hospital in Brisbane were asked to participate in the study; 98 % of those attending the booking in clinic were enrolled in the study and 6753 women who gave birth to 7223 children that were not adopted out before leaving the hospital, constitute the MUSP birth cohort. At the 14- and 21-years follow-ups, the offspring completed health, social and lifestyle questionnaires. Details of the MUSP birth cohort including recruitment of participants and measurements taken at each follow-up visit have been previously published^(30)^.

This current study examines data from the 21-year follow-up (2001–2003) of the offspring. A total of 3805 (52·7 %) offspring, young adults from this point forward, responded to a core questionnaire and 3654 (1731 men; 1923 women) completed a food frequency questionnaire (FFQ)^(30,31)^. A core questionnaire addressed the following topics: sociodemographic characteristics; lifestyles; general health and well-being; and other topics such as psychosocial and mental health of respondents. Exclusions (total *n* 894) were based on prior work with this cohort^(31)^. We excluded 429 young adults with incomplete FFQ information, such as a missing or blank FFQ page or whole section, or which had a lack of information on alcohol consumption. Another 465 young adults were excluded because of implausible energy intake reports that are defined as <500 or ≥3500 kcal/d for female participants and <800 or ≥ 4000 kcal/d for male participants^(31)^. We only included participants with <40 % missing FFQ items (blanks), leaving an analytical cohort of 2760 young adults with useable dietary data, out of which 2665 (1135 men, 1530 women) had complete data for each variable included in the current analyses (see online Supplemental Fig. 1).

### Assessment of lifestyles

In this study, we consider five measures of lifestyle, namely smoking status, alcohol consumption, physical activity and sedentary/screen-based activity as well as BMI based on availability of the data and previous literature evaluating possible associations of some of these variables with dietary patterns among young adults^(18–25)^. Information on each lifestyle measure comprised a self-report provided by young adults.

Smoking status was measured as the number of cigarettes per day in three categories; non-smoker, 1–19 cigarettes (light smokers) or ≥ 20 cigarettes (heavy smokers). Alcohol consumption was measured based on estimates of the mean number of standard drinks consumed/d; abstainer (0), light consumer (0·1–0·5), moderate consumer (0·51–1), heavy consumer (1·01–3·4) or very heavy consumer (≥ 3·5). This latter variable was aggregated into four categories as abstainers, and light, moderate and heavy consumers, for further analysis.

Further, participants were asked about their physical activity using questions from the Active and Inactive Australians Questionnaire (AIAQ)^(32)^. The AIAQ questionnaire includes questions about vigorous exercise, less vigorous exercise and walking along with average weekly sessions (‘never’, ‘1–2 times’ and ‘3 or more times’) of engagement in each type of activity during the past 6 months^(32)^. Exercise was considered vigorous when it made a participant breath harder or puff and pant including such activities as swimming, tennis, netball, athletics and running and less vigorous when it was for recreation purpose including such activities as bike riding and dancing. We quantified categories of sessions and summed up these values to obtain a weekly average number of sessions that a participant was engaged in any type of physical activity^(33)^. Participants were then grouped as physically inactive (0 sessions), insufficient (1–5 sessions) and sufficient (≥ 5 sessions) physical activity for health based on Active Australia Survey guideline^(34)^.

In addition, participants were assessed for sedentary/screen-based activity, defined by time spent watching television (TV), using daily number of hours they spent watching TV (weekend days and weekdays) separately with the following response options: ‘never watched’ and watched for ‘<1 h’, ‘1–3 h’, ‘3–5 h’, ‘5–7 h’ and ‘7 h or more’. We quantified categories of each response to estimate the average daily time spent watching TV^(33)^. First, we calculated the average values of time spent on weekdays and on weekend days and multiplied by five and two, respectively. We summed up these values and divided them by seven to obtain daily average time spent watching TV for each participant. Participants were then grouped into three categories as <2 h/d (bottom 25 %), 2–4 h/d (middle 50 %) or > 4 h/d (top 25 %) duration of time spent watching TV based on the relative distribution of daily average time spent for this purpose.

BMI (kg/m^2^) was calculated separately from measured (*n* 1967) and self-reported (*n* 2681) weight (kg) and height (m) of participants and categorised into three groups based on WHO criteria; normal weight (<25 kg/m^2^), overweight (25–30 kg/m^2^) or obese (>30 kg/m^2^)^(35)^. We found a strong correlation (*r* = 0·98; *P* < 0·0001) between BMI calculated from measured and self-reported data. This finding is similar to a report from a nationally representative sample of adult Australians^(36)^. Hence, we used BMI calculated from self-reported data to maintain a larger cohort (Table 1). Arguably, people may choose to consume healthy foods to maintain their normal body weight or they may modify their food choices when they become obese in order to reduce their body weight. Thus, we considered BMI as one of the modifiable lifestyle variables that could influence dietary intakes of persons^(37,38)^.

**Table 1:** General characteristics among 2665 young adults (18–23 years) in Australia (2001–2004)

Characteristics*	Total (*n* 2665)	Men (*n* 1135)	Women (*n* 1530)	*P*-value
	%	%	%	
Categorical variables				
Smoking intensity (cigarettes/d)				
Non-smokers	66·2	63·26	68·30	<0·0001
Smokers 1–19	29·2	30·13	28·50	
Smokers ≥ 20	4·7	6·61	3·20	
Alcohol consumption (standard drinks/d)				
Non-consumers (0)	5·9	27·58	49·08	<0·0001
Light consumers (0·1–0·5)	39·9	5·29	6·41	
Moderate consumers (0·51–1)	18·9	18·68	19·02	
Heavy consumers (1·01–≥ 3·5)	35·3	48·46	25·49	
Physical activity (sessions/week)				
Sufficient (≥ 5)	33·9	33·04	34·44	0·6808
Insufficient (1–4·99)	56·7	57·71	56·01	
Sedentary (0)	9·4	9·25	9·54	
Time spent watching TV (h/d)				
<2 h	23·8	22·56	24·71	0·1029
2–4 h	43·4	42·47	44·12	
≥4 h	32·8	34·98	31·18	
BMI (kg/m^2^) category				
Normal (<25)	67·8	65·81	69·28	0·0124
Overweight (25–30)	20·7	23·35	18·69	
Obese (≥ 30)	11·5	10·84	12·03	
Level of income (AUD/week)				
High income (> 450)	37·1	45·99	30·52	<0·0001
Middle income (180–450)	41·7	34·89	46·67	
Low income (<180)	21·2	19·12	22·81	
Level of education				
Post-secondary school	27·5	23·88	30·13	<0·0001
Complete secondary school	55·1	55·42	54·90	
Incomplete secondary school	17·4	20·70	14·97	
Marital status				
Not married†	78·9	87·93	72·22	<0·0001
Married or living together	21·1	12·07	27·78	
Living arrangements				
Living with parents	60·2	69·08	53·6	<0·0001
Not living with parents	39·8	30·92	46·4	
Vitamin supplements use				
No	67·4	76·48	60·85	<0·0001
Yes	32·6	23·52	39·15	
Continuous variables				
Age, years				
Mean	20·6	20·6	20·6	0·5750
sd	0·84	0·85	0·83	
Energy, kcal				
Mean	2022·1	2473·3	1687·4	<0·0001
sd	766·1	751·5	583·7	
Fat, E %				
Mean	36·8	37·8	36·1	<0·0001
sd	5·2	4·8	5·4	
Carbohydrate, E %				
Mean	42·0	40·5	43·2	<0·0001
sd	6·1	5·7	6·2	
Protein, E %				
Mean	20·0	20·1	20·0	0·4157
sd	3·0	2·9		

E % is percent energy.

* Values are in % or mean ± sd.

† Not married includes never married and widowed, divorced and separated.

### Assessment of covariates

A number of sociodemographic characteristics including living arrangements have been associated with dietary patterns^(20–23,31,39)^, whereas dietary supplement use has been associated with the adoption of other healthy habits such as a healthier diet, better physical activity, avoiding smoking and alcohol consumption and healthy body weight^(40)^. We used self-reports of young adults to obtain information on sociodemographic measures. These measures include age (in years), gender, level of education (incomplete secondary school, secondary school, College-Tafe and University), youth income (weekly) in AU$ (no income, 1–39, 40–79, 80–119, 120–159, 160–199, 200–299, 300–399, 400–499, 500–599, 600–699, 700–799, 800–999, 1000–1499 or ≥ 1500), marital status (never married, live together, married, separated-divorced-widowed) and living arrangements (living with *v*. not with family). Income was regrouped into three categories: low income (bottom 25 %); middle income (middle 50 %) and high income (top 25 %) based on relative distribution of average income^(33)^. Marital status and levels of education were aggregated into two and three categories, respectively. Also, information on vitamin supplement use (yes/no) was obtained by self-reports of young adults (Table 1).

### Assessment of dietary intake

Usual dietary intake was assessed using the Cancer Council of Victoria’s semiquantitaive and validated 101-food items FFQ^(41,42)^. Relative validity of nutrient intake for this FFQ has been documented, and the FFQ was found to be useful in the assessment of habitual intake in the Australian adult population^(42)^. Correlation coefficients for energy-adjusted nutrient intake between 7-d weighed food records and the FFQ ranged from 0·28 (vitamin A) to 0·78 (carbohydrate)^(42)^. Study participants were asked to report on how often they usually consumed a specified food item (on a 10-point scale from ‘never’ to ‘3 or more times a day’) over the previous 12 months, including six items on the consumption of alcoholic beverages. These were converted to daily equivalents for statistical analysis. The FFQ also included 10 short questions on the consumption of fruit, vegetables, sugar, eggs, and the quantity and type of milk, cheese, bread and fat spreads. Photographs of different portion sizes of selected food items and dishes were included in the FFQ to assist in the calculation of daily energy and nutrient intake. These were estimated with the use of software developed by the Cancer Council of Victoria on the basis of Australian food composition table as contained in the Nutrient Data Table for Use in Australia (NUTTAB95), the national government food composition database of Australian foods^(43)^.

### Derivation of dietary patterns

To derive dietary patterns, first the 95 food items (excluding six items on alcohol intake) were grouped into 33 food groups on the basis of the similarity of food type, nutrient composition or culinary usage to reduce within person variation in dietary intake. Individual food items that constituted a distinct item (e.g. eggs, butter, pizza) or those thought to represent a particular eating pattern (e.g. potato, fruit juice, sugar) on their own were preserved (Table 2). We computed the average intake (in grams) of each participant’s food group by summing the intake of the individual foods that made up each food group.

**Table 2:** Food groupings used in dietary pattern analysis among 2665 young adults (18–23 years) in Australia (2001–2004)

Food or food group (g/d)	Food items
High-fat milk	Full cream milk; yoghurt, icecream, flavoured milk drink
Low-fat milk	Reduced fat milk; skim milk
Low-fat cheese	Low fat cheese, ricotta or cottage cheese
High-fat cheese	Hard cheese; firm cheese; soft cheese; cream cheese
Butter	Butter
Spreads containing fat	Margarine; polyunsaturated margarine; monounsaturated margarine; butter and margarine blends
Eggs	Boiled, fried, and scrambled eggs
Poultry	Chicken with or without skin
Red meat	Beef; veal; lamb; pork
Processed meat	Sausages or frankfurters; hamburger; ham; bacon; corned beef, luncheon meats or salami
Fish, other	Steamed, grilled or baked fish; tinned fish (salmon, tuna, sardines, etc.)
Fish, fried	Fried fish (includes take away)
Citrus fruit	Orange or other citrus fruit
Other fruit	Tinned or frozen fruit (any kind); strawberries; apples; pears; bananas; pineapple; watermelon, rockmelon, honeydew etc.; apricots; peaches or nectarines; mango or paw paw; avocado
Fruit juice	Fruit juice
Vegetables	Lettuce, endive, or other salad greens; Silverbeet or spinach; onion or leeks; beetroot; carrots, pumpkin, peppers (capsicum); broccoli; cauliflower; cabbage or Brussel sprouts; cucumber; celery; garlic; mushrooms; zucchini
Tomatoes	Tomato sauce, tomato paste or dried tomatoes; fresh or tinned tomatoes;
Potatoes	Roasted or fried (include hot chips), potatoes cooked without fat
Legumes	Bean sprouts; baked beans; soy beans, soy bean curd, tofu; other beans (chick peas, lentils etc.); peas; green beans; soy milk
Cereal products	Crackers, crispbreads, dry biscuits
Other bread	High fibre white bread, refined white bread
Wholemeal bread and whole grains	Wholemeal bread, rye bread, multigrain bread
Breakfast cereals	All bran, sultana bran, fibreplus, branflakes; weet bix, vita brits, weeties; cornflakes, nutrigrain, special K; porridge; muesli
Rice	Rice (all type)
Pasta	Pasta or noodles (includes lasagne)
Pizza	Pizza
Meat pies and savoury pastries	Meat pies, pastries, quiche and other savoury pastries
Snacks	Maize chips, potato crisps, twisties
Chocolate	Chocolates
Cakes and Pastries	Cakes, sweet pies, tarts, and other sweet pastries; sweet biscuits
Sugar	Sugar
Nuts	Nuts
Non-fat spreads	Jam, marmalade, honey, syrup; Vegemite, Marmite, Promite; Peanut butter

Dietary patterns were derived applying factor analysis with principal component extraction (PROC FACTOR in SAS) based on the consumption of 33 predefined food groups. The factor scores obtained were rotated by orthogonal transformation to achieve uncorrelated and more interpretable structures^(44)^. We considered solutions containing 2–4 factors. After examining factor solutions with eigenvalues > 1·25, we chose a two-factor solution based on the break point of scree plot and interpretability of the factors^(44)^. We identified two main dietary patterns and named them based on food groups with high loadings^(45)^. Food groups with absolute value of factor loadings ≥ 0·3 were considered as meaningfully contributing to each dietary pattern^(46)^. Inter-item reliability for foods groups with high loadings in each dietary pattern was assessed by Cronbach’s *α* coefficients. Factor scores for each dietary pattern were calculated by summing the products of intakes of food groups weighted by loading coefficients^(46)^ as have been presented in Table 3.

Initially, we conducted dietary pattern analysis for men and women separately. The gender-specific dietary patterns identified were similar in relation to the number of factors identified and the foods that loaded highly. Therefore, the combined cohort of men and women was used for the present analysis. Because alcohol intake was included in the analyses as a lifestyle correlate of dietary patterns, we excluded items measuring alcohol intake as these contribute to derive dietary patterns.

### Statistical analyses

Normality of the continuous variables including standardised scores for each dietary pattern and computed food groups values (g/d) used to drive dietary patterns and age (in years) was checked visually using Q–Q plots of residuals against the predicted values, histograms and observing the skewness and kurtosis measures of their respective standard errors. Each of the dietary pattern scores and age were approximately normally distributed. However, computed food groups had positively skewed distributions; hence, their values were log-transformed before deriving dietary patterns. The standardised values of the two derived dietary pattern scores were considered as separate outcomes, and multivariable linear regression analysis was used for the main analyses. Differences in continuous and categorical variables between males and females were compared using the independent sample t-test and chi-square, respectively.

We first performed bivariate analyses to identify candidate variables for multivariable models using significance level of <0·20. All explanatory variables had *P*-values <0·20 for the Western dietary pattern and hence, all of them were entered into a series of multivariable model. Despite having *P*-values > 0·20 in bivariate analyses for a prudent dietary pattern, living arrangements and gender of participants were maintained in multivariable model based on previous findings^(28,31,39)^. Before fitting multivariable models for each dietary pattern, we examined multicollinearity among the explanatory lifestyle variables with the use of the variance inflation factor and Pearson correlation coefficients. Further, we examined evidence of interaction terms between gender and each lifestyle correlate examined. There was no evidence of collinearity or interaction observed in the current analysis. Thus, all analyses were based on the entire cohort of men and women.

To identify lifestyle variables significantly correlated with dietary patterns of young adults, a series of multivariable linear regression models were fitted separately for each dietary pattern adjusting for covariates including sociodemographic variables and energy intakes. The first two models were presented in Supplemental Table 1. In the first model, we run a model containing explanatory lifestyle variables including levels of smoking, alcohol consumption, physical activity, watching TV and BMI category. In the second model, we adjusted for covariates including age in years, gender, income, education, marital status, living arrangements and vitamin supplement use. In the fully adjusted model, we additionally adjusted for total energy intake of respondents (Table 4). All tests were two-sided and *P*-value <0·05 was considered statistically significant. The results are mean differences in dietary patterns scores across levels of examined lifestyle correlates and reported as standardised and unstandardised beta coefficients (*β*s) with their 95 % CIs presented in Table 4 and Supplemental Table 2, respectively. Also, standardised *β*s are presented in Supplemental Table 2. All analyses were performed using SAS for window (version 9.4; SAS Institute).

**Table 3:** Factor loadings of the principal dietary patterns identified among 2665 young adults (18–23 years) in Australia (2001–2004)*

Food/food group	Western dietary pattern	Prudent dietary pattern
High-fat milk	0·51	
Low-fat milk	−0·46	0·26
Low-fat cheese	−0·41	
High-fat cheese	0·41	
Spreads containing fat	0·33	
Eggs	0·22	0·20
Poultry	0·43	0·29
Red meat	0·56	0·24
Processed meat	0·70	
Fish, other	0·19	0·44
Fish, fried	0·52	0·22
Citrus fruit		0·32
Other fruit		0·53
Fruit juice		0·38
Vegetables		0·49
Tomatoes		0·33
Potatoes		0·30
Legumes		0·40
Cereal products		0·49
White bread	0·52	−0·32
Wholemeal bread	−0·42	0·40
Breakfast cereals		0·43
Rice, all type		0·47
Pasta or noodles	0·25	0·47
Pizza	0·56	
Meat pies and savory pastries	0·55	
Snacks	0·59	
Chocolate	0·33	0·21
Cakes and pastries	0·44	0·30
Sugar	0·26	
Nuts		0·43
Non-fat spreads	0·23	0·45
Explained variance (%)	14·11	9·23
Cronbach’s *α*	0·77	0·70

* Absolute values <0·20 for each factor loadings were not shown for simplicity.

As a sensitivity analysis, we fitted separate models to test if total effect of a lifestyle variable of interest out of four of these variables, namely physical activity, watching TV, smoking and alcohol consumption included in this study changes after successively leaving three or fewer others out of a fully adjusted model (Table 4) to have estimates for each from models with different covariate subsets. These sensitivity analyses did not substantially alter the estimates. Thus, we interpret total effects from a fully adjusted model above containing all lifestyle variables adjusted for covariates^(47)^.

## Results

### Characteristics of the study participants

Data from a total of 2665 young adults was used for this analysis. Sociodemographic and lifestyle characteristics are presented in Table 1. Mean ± sd age of participants was 20 ± 0·8 years (range 18–23 years). A majority of the participants were females (*n* 1530; 57·3 %) and not married (72·9 %). A higher proportion of participants completed secondary school (55·1 %) and were in the middle-income (41·7 %) group. Two-thirds of participants were non-smokers, while the majority of smokers smoked fewer than 20 cigarettes a day. A majority of the participants were light (39·9 %) and heavy (35·3 %) consumers of alcohol. A majority of the participants had insufficient (1–5 sessions a week) physical activity and viewed TV for 2–4 h/d, respectively. Nearly two-thirds of participants were living with their parents and reported using no vitamin supplements, while nearly one-third were overweight or obese (BMI ≥ 25 kg/m^2^).

Differences in sociodemographic and lifestyle characteristics are also presented by sex. Compared to males, female participants are more likely to be poor, not married, not living with parents, obese, non-users of vitamin supplements and less likely to be overweight and heavy smokers and alcohol consumers. Regarding macronutrient intakes, males had a higher total energy intake and higher percent energy from fat, while females had a higher percent energy from carbohydrate; there was no significant difference in energy intake from protein between males and females (Table 1).

### Characterising dietary patterns

Using a total cohort of men and women, we identified two major dietary patterns, the Western and prudent patterns that collectively explained 21·6 % (13·0 % and 8·6 %, respectively) of variation in dietary intakes in young adults. The Western pattern was characterised by high consumption of red meats, processed meat, poultry, fried fish, pizza, meat pies and savoury pastries, high-fat dairy products, fat containing spreads, white bread, pasta, snacks, chocolate and cakes and pastries and low consumption of low-fat dairy products and wholemeal bread. The prudent pattern was characterised by high consumption of vegetables, fruit, fish, potatoes, legumes, cereals, wholemeal bread, rice, pasta, nuts and non-fat containing spreads and low consumption of white bread. Inter-item reliability in each dietary pattern was assessed by Cronbach’s *α* coefficients and these were 0·74 for each pattern. Factor loadings for each dietary pattern are presented in Table 3. The standardised factor scores for the Western dietary pattern ranged from −3·46 to 2·56 and was from −3·21 to 3·11 for a prudent pattern. The lower and higher dietary pattern scores suggest lesser and greater adherence to a particular dietary pattern.

**Table 4:** Standardised regression coefficients (95 % CI) in fully adjusted models according lifestyle factors among 2665 young adults (18–23 years) in Australia (2001–2004)*

Characteristics	Prudent pattern			Western pattern		
	*β*	95 % CI	*P*-value	*β*	95 % CI	*P*-value
Physical activity (sessions/week)						
Sufficient (≥ 5)	Reference			Reference		
Insufficient (1–4)	−0·12	−0·15, −0·09	<0·0001	0·14	0·11, 0·17	<0·0001
No physical activity (0)	−0·14	−0·17, −0·11	<0·0001	0·11	0·08, 0·14	<0·0001
Time spent watching TV (h/d)						
<2 h	Reference			Reference		
2–4 h	−0·06	−0·09, −0·02	0·0037	0·07	0·04, 0·11	<0·0001
≥ 4 h	−0·12	−0·16, −0·09	<0·0001	0·11	0·07, 0·14	<0·0001
Smoking (cigarettes/d)						
Non-smokers	Reference			Reference		
Smokers 1–19	−0·05	−0·09, −0·02	0·0007	0·06	0·03, 0·08	<0·0001
Smokers ≥ 20	−0·08	−0·12, −0·05	<0·0001	0·05	0·02, 0·08	0·0005
Alcohol consumption (standard drinks/d)						
Non-consumers (0)	−0·02	−0·05, 0·01	0·2902	−0·003	−0·03, 0·02	0·8449
Light consumers (0·1–0·5)	Reference			Reference		
Moderate consumers (0·51–1)	0·03	−0·01, 0·06	0·1251	0·01	−0·02, 0·04	0·5277
Heavy consumers (1·01–≥3·5)	−0·03	−0·06, 0·01	0·1037	0·04	0·01, 0·07	0·0095
BMI (kg/m^2^) category						
Normal (< 25)	Reference			Reference		
Overweight (25–30)	0·04	0·01, 0·07	0·0163	−0·04	−0·07, −0·02	0·0014
Obese (≥ 30)	0·04	0·01, 0·07	0·0169	−0·03	−0·06, −0·01	0·0191

* Models for each dietary pattern were run with the following lifestyle variables included: smoking, alcohol consumption, physical activity, time spent watching TV and BMI category.

All results are adjusted for age in years, gender, income, education, marital status, living arrangement, vitamin supplement use and total energy intake.

### Lifestyle correlates of dietary patterns

Table 4 presents lifestyle correlates of each dietary pattern fully adjusted for age, gender, income, education, marital status, living arrangements, vitamin supplement and total energy intakes. The strongest lifestyle correlates of both a prudent and the Western dietary patterns are measures of physical activity, time spent watching TV and smoking in each fully adjusted model. Participants with a greater adherence to a prudent dietary pattern are less likely to engage in lower levels of physical activity, are less likely to watch TV for extended periods of time and are less likely to be light and heavy smokers. There was also a weaker but significant positive association of a prudent dietary pattern with BMI of young adults; those with a greater adherence to this pattern are more likely to be overweight and obese. Alcohol consumption was not associated with a prudent dietary pattern.

For each lifestyle variable correlated with a prudent dietary pattern, a similar strength but opposite direction of association with the Western dietary pattern is observed. Young adults who adhere more to the Western dietary pattern are more likely to engage in lower levels of physical activity, are more likely to watch TV for extended periods of time and are more likely to be light and heavy smokers. Alcohol consumption and BMI were weakly associated with the Western dietary pattern. Those with a greater adherence to the Western pattern are more likely to be heavy consumers of alcohol and are less likely to be overweight and obese (Table 4).

We also evaluated the associations between dietary pattern scores and energy and energy adjusted nutrient intakes of participants. The two dietary patterns differ with the Western pattern being positively and a prudent pattern being negatively associated with percent energy from fat and intakes of total fat, saturated fat, monounsaturated fat, total cholesterol and sodium. By contrast, the Western pattern is negatively and a prudent pattern is positively associated with percent energy from carbohydrate and protein and intakes of protein, carbohydrate, dietary fibre, sugar, starch, total beta-carotene and selected vitamins and minerals (see online Supplemental Table 3).

## Discussion

We have identified two main dietary patterns, the Western and prudent patterns in this large study of young adults aged 18–23 years. The Western pattern is correlated with higher consumption of processed and red meat, poultry, high-fat dairy products, refined grains, takeaway foods, fried fish, fat and non-fat spreads and snacks and sweets, whereas a prudent pattern is correlated with higher consumption of fruit, vegetables, potatoes, legumes, nuts, whole grains, fish, cereals and non-fat spreads. The two dietary patterns explained 21·6 % of the variance in dietary intake in this study. This is consistent with findings from studies involving young adults from Brazil and the UK^(21,23)^. Studies of young adults in the USA and in Canada have reported higher (31 %) and lower (15·6 %) total variance in food intakes explained in the dietary patterns that they have identified^(19,25)^.

We labeled the dietary patterns identified in this study based on previous literature^(45)^. However, these dietary patterns are slightly different from those reported in other studies because food group ‘cakes and pastries’ is correlated with both patterns in this study. Multiple loadings of food items have been observed in previous studies^(22)^.

Different studies report varying numbers and types of dietary patterns in nutritional epidemiology. Consistent with our results, three studies of young adults have identified similar types of dietary patterns to those reported in the current study^(19,22,24)^. One study among US young adults has identified Western and prudent dietary patterns^(19)^. A study from Denmark has identified traditional Western and green dietary patterns in young adults^(22)^, with comparable food composition to the Western pattern and prudent pattern in this study. Finally, a study of young women in the UK has identified the prudent and high-energy dietary patterns, the latter pattern having similar characteristics to the Western pattern observed in this study^(24)^. Moreover, studies involving adult women from the “Mamma & Bambino” cohort in Italy have identified the Western and prudent dietary patterns with similar food composition to those observed in this study^(48,49)^. However, studies in young adult populations in Brazil and UK have identified up to five dietary patterns with varying food compositions^(20,21,23)^. A study involving young (25–30 years) and middle-aged (50–55 years) women in Australia has identified up to six dietary patterns composed in various ways^(37)^. The variations in types of dietary patterns identified using data-driven methods (e.g. principal components analysis) may reflect variations of foods included in the specific studies, the distribution of these foods in the data set and inconsistencies among the studies in defining and interpreting dietary patterns^(50,51)^. The variations in dietary patterns among studies using data-driven methods to define dietary patterns could arise from differences in methods used to assess dietary intake as well as differences in food grouping and the number of food items and food groups used in the statistical analysis^(52)^. To address these inconsistencies, researchers have suggested looking at the results of individual studies to ascertain which foods have substantially contributed to the dietary patterns of interest^(50,51)^.

The current study suggests that dietary patterns are associated with the broader lifestyles of young adults. Lower levels of physical activity, extended duration of watching TV and smoking were the strongest correlates of each dietary pattern. In this study, participants with lower levels of physical activity had less adherence to a prudent dietary pattern, while they had greater adherence to the Western pattern. Other studies have also reported that more active participants had higher scores for healthier dietary patterns and lower scores for less healthy dietary patterns in young men and women in Brazil and the USA, as well as in a study of young and middle-aged women in Australia^(19,20,37)^. However, the study in the USA found no association between physical activity and a prudent dietary pattern with this latter null finding in the USA study possibly explained by a relatively larger sample size in this study^(19)^. We have found that participants with longer duration of watching TV had lower adherence to a prudent dietary pattern and a higher adherence to the Western pattern. A few studies involving young women in the UK and university students in the USA have reported no association between duration of watching TV and any of the identified dietary patterns^(18,24)^. This could be explained by the fact that both studies had relatively small samples and may have been underpowered to detect a difference. However, a study in Brazilian adolescents found a positive association between eating while watching TV and an unhealthy dietary pattern, partly supporting the findings observed in the current study^(53)^. Results of this study suggest young adults with lower levels of physical activity and longer duration of watching TV are less likely to consume healthy foods rich in micronutrients and fiber, and they are more likely to consume unhealthy foods rich in fat and dietary cholesterol as has been reported in other studies^(7,16,17)^.

In this study, smokers were less likely to adhere to a prudent dietary pattern and more likely to adhere to the Western pattern. Our findings are consistent with previous studies of young adults in Brazil and in the USA, as well as with a study of young and middle-aged women in Australia^(19,20,37)^. Evidence from previous studies of young adults shows that smoking has been associated with reduced intakes of fruits, essential fatty acids, fiber and micronutrients and higher intakes of energy, total fat, saturated fat and dietary cholesterol^(14,15)^.

In this study, heavy consumption of alcohol was positively associated with the Western dietary pattern, whereas it showed no association with a prudent pattern. Our findings in part are consistent with findings of a study involving a pooled cohort of young and middle-aged women in Australia^(37)^. Higher consumption of alcohol has been associated with lower consumption of healthy foods and higher consumption of unhealthy foods among Spanish university students^(13)^. A study in the US young adults found no association between alcohol consumption, and the Western and prudent dietary patterns^(19)^, partly supporting our findings. The observed different results between the US study and the current study might reflect the relatively lower study power in the US study.

Unexpectedly, we observed that obese and overweight participants had respective higher and lower scores for the prudent and the Western dietary patterns. Studies of young women and university students in the UK observed no association between BMI and any of the identified dietary patterns^(23,24)^. These studies in the UK had small samples, possibly explaining the observed differences^(23,24)^. Our findings are consistent with a study involving young and middle-aged women in Australia^(37)^. The results of this study could suggest that people may select healthy foods when they become obese or overweight^(38)^. Also, underreporting and omitting snacks are common problems in accurately assessing dietary intakes of general population, and more so in obese and overweight people, partly explaining the current findings^(54,55)^.

Notably, a number of lifestyle variables in the fully adjusted model are correlated and it is arguable which of these effects precedes the other in this cross-sectional study. For example, physical activity could arguably reduce time spent watching TV or watching TV could, arguably, reduce the time available for physical activity. It could be suggested that some lifestyle variables in the fully adjusted model could be treated as moderators in assessing their associations with dietary patterns. These considerations while most relevant in longitudinal study designs are not central to our argument. Our aim has been to establish whether dietary patterns are associated with and hence, part of the more general aspect of the lifestyles of respondents. Furthermore, in sensitivity analyses, removing lifestyle variables which are arguably moderators from fully adjusted models (e.g. physical activity in the association between time spent watching TV and dietary patterns or vice versa) has no observable impact on the broader associations we have observed.

### Strengths and limitations of the study

Our study has several strengths. We used data from a community-based MUSP birth cohort and we had a relatively large sample size included in the analyses. Also, we used dietary data from a validated FFQ that has been designed for use in adult population in Australia^(42)^. Observing the clustering of dietary and other habits of lifestyle in young adults is another strength of our study, given young adulthood is arguably a period in the life course during which a range of lifestyle may develop including habits of dietary intake, physical activity, sedentary activity, cigarrete smoking and alcohol consumption. Furthermore, this study, to our knowledge, is the first to use data-driven method to assess dietary patterns of both young men and women in Australia.

Some limitations of this study are acknowledged. First, our study used a cross-sectional data from the MUSP cohort during the 21-year follow-up (2001–2004) and hence, causality in the observed associations cannot be ascertained. The narrow age range (18–23 years) of respondents in our sample is acknowledged. We have noted that some data was excluded due to some missing food items in the FFQ and implausible energy intake which might have affected some estimates in our study.

We have used data that have been collected during 2001–2004 and our findings might not reflect dietary pattern of the current generation. There have been declines in the availability and intake of added or refined sugars in adult men (such change in women was NS) and sugar-sweetened beverages in men and women adults over the period of 1980/1995–2011 in Australia^(56,57)^. Also, there has been improved consumption of healthy foods such as fruit, vegetables, nuts and whole grain cereals during 1995–2011 in Australian adults^(57)^. However, more recent data indicate the majority of adult Australians, particularly in the younger age group, are failing to meet national dietary guidelines. Only 24 % of women and 15 % of men have dietary intakes of both the fruit and vegetable consistent with national guidelines^(58)^.

There has been substantial loss to follow-up in the MUSP cohort, a long-term follow-up study of mothers and their offspring for more than 30 years. Those disproportionately lost to follow-up are more likely to involve respondents who are young and socially disadvantaged, of separated or divorced marital status and have higher rates of unhealthy lifestyles^(30,31)^. Loss to follow-up affects sample means, but they rarely affect estimates of association^(59,60)^. Similar estimates of association in the groups retained in the study and those lost to follow-up are reported in a study that has evaluated the impact of biased loss to follow-up using the MUSP birth cohort data^(60)^. The MUSP birth cohort is not representative of the entire Australian population, but the cohort comprises a broad cross-section of the population^(31)^.

## Conclusion

Our study has identified two main dietary patterns among young adults, namely the Western and prudent patterns. The lifestyles of young adults including physical activity, watching TV, smoking and alcohol consumption are associated with these dietary patterns. These findings suggest that there is clustering of lifestyle features associated with the main patterns of food consumption observed in a developed economy like Australia. This clustering involves a number of lifestyle features such as physical activity, sedentary activity, cigarette smoking and alcohol consumption. This clustering of factors raises three important issues. Firstly, from a research perspective any association between patterns of food consumption and health outcomes needs to take account of this clustering. Secondly, from a clinical or therapeutic perspective, efforts to modify diet in young adults need to include a focus on the multiple lifestyles of those whose patterns of food consumption predisposes them to a range of adverse health outcomes. Thirdly, from policy perspective, because a range of lifestyles are partly formed during young adulthood, nutrition and health policies should target emerging adults to promote healthy lifestyle and subsequently reduce the risk of unhealthy lifestyle related adverse health outcomes at later ages. Finally, evaluating the contributing factors to the development of unhealthy lifestyles and their clustering over time needs to be determined using prospective cohort studies.

## Supplementary material

For supplementary material accompanying this paper visit https://doi.org/10.1017/S1368980021003864. click here to view supplementary material
