# Supplementary material for: Lifestyle correlates of dietary patterns among young adults: evidence from an Australian birth cohort
Source: Public Health Nutr. 2021 Sep 6;25(8):2167–78. doi: 10.1017/S1368980021003864 (PMC9991692; doi:10.1017/S1368980021003864)
Supplement: Supplementary file 1 [file S1368980021003864sup001.docx]

**Supplementary Table 1:** Standardised regression coefficients (95% CI) in fully adjusted models according lifestyle factors among 2665 young adults (18-23 years) in Australia (2001-2004)^1 2^

| **Characteristics** | **Prudent pattern** |  | **Western pattern** |  |
| --- | --- | --- | --- | --- |
|  | **β (95% CI)** | ***P*-value** | **β (95% CI)** | ***P*-value** |
| **Model 1** |  |  |  |  |
| Physical activity (sessions/wk) |  |  |  |  |
| Sufficient (≥5) | Reference |  | Reference |  |
| Insufficient (1-4) | -0.14 (-0.18, -0.10) | <.0001 | 0.14 (0.10, 0.17) | <.0001 |
| No physical activity (0) | -0.17 (-0.21, -0.13) | <.0001 | 0.10 (0.06, 0.14) | <.0001 |
| Time spent watching TV (h/d) |  |  |  |  |
| < 2 h | Reference |  | Reference |  |
| 2-4 h | -0.01 (-0.06, 0.03) | 0.5698 | 0.12 (0.08, 0.17) | <.0001 |
| ≥ 4 h | -0.08 (-0.13, -0.04) | 0.0005 | 0.18 (0.13, 0.23) | <.0001 |
| Smoking (cigarettes/d) |  |  |  |  |
| Non-smokers | Reference |  | Reference |  |
| Smokers 1-19 | -0.06 (-0.10, -0.02) | 0.0016 | 0.07 (0.03, 0.11) | 0.0002 |
| Smokers ≥20 | -0.10 (-0.14, -0.06) | <.0001 | 0.06 (0.03, 0.10) | 0.0010 |
| Alcohol consumption (standard drinks/d) |  |  |  |  |
| Non-consumers (0) | -0.01 (-0.04, 0.03) | 0.7792 | 0.02 (-0.02, 0.06) | 0.3561 |
| Light consumers (0.1-0.5) | Reference |  | Reference |  |
| Moderate consumers (0.51-1) | 0.06 (0.02, 0.10) | 0.0025 | 0.07 (0.03, 0.11) | 0.0004 |
| Heavy consumers (1.01-≥3.5) | 0.05 (0.00, 0.09) | 0.0342 | 0.19 (0.14, 0.23) | <.0001 |
| BMI (kg/m^2^) category |  |  |  |  |
| Normal (<25) | Reference |  | Reference |  |
| Overweight (25-30) | 0.03 (-0.01,0.07) | 0.1377 | -0.03 (-0.07, 0.00) | 0.0764 |
| Obese (≥30) | 0.02 (-0.02, 0.05) | 0.4258 | -0.05 (-0.09, -0.01) | 0.0112 |
| **Model 2** |  |  |  |  |
| Physical activity (sessions/wk) |  |  |  |  |
| Sufficient (≥5) | Reference |  | Reference |  |
| Insufficient (1-4) | -0.14 (-0.18, -0.10) | <.0001 | 0.12 (0.09, 0.16) | <.0001 |
| No physical activity (0) | -0.16 (-0.20, -0.12) | <.0001 | 0.09 (0.05, 0.12) | <.0001 |
| Time spent watching TV (h/d) |  |  |  |  |
| < 2 h | Reference |  | Reference |  |
| 2-4 h | -0.01 (-0.06, 0.04) | 0.6988 | 0.12 (0.08, 0.16) | <.0001 |
| ≥ 4 h | -0.07 (-0.12, -0.02) | 0.0035 | 0.16 (0.12, 0.20) | <.0001 |
| Smoking (cigarettes/d) |  |  |  |  |
| Non-smokers | Reference |  | Reference |  |
| Smokers 1-19 | -0.04 (-0.08, 0.00) | 0.0432 | 0.07 (0.04, 0.11) | <.0001 |
| Smokers ≥20 | -0.10 (-0.14, -0.06) | <.0001 | 0.03 (0.00, 0.07) | 0.0622 |
| Alcohol consumption (standard drinks/d) |  |  |  |  |
| Non-consumers (0) | -0.01 (-0.05, 0.03) | 0.7056 | 0.01 (-0.03, 0.04) | 0.6997 |
| Light consumers (0.1-0.5) | Reference |  | Reference |  |
| Moderate consumers (0.51-1) | 0.05 (0.01, 0.09) | 0.0147 | 0.03 (0.00, 0.07) | 0.0727 |
| Heavy consumers (1.01-≥3.5) | 0.01 (-0.03, 0.05) | 0.6613 | 0.08 (0.04, 0.12) | <.0001 |
| BMI (kg/m^2^) category |  |  |  |  |
| Normal (<25) | Reference |  | Reference |  |
| Overweight (25-30) | 0.03 (-0.01, 0.06) | 0.1625 | -0.05 (-0.09, -0.02) | 0.0019 |
| Obese (≥30) | 0.02 (-0.01, 0.06) | 0.2225 | -0.05 (-0.08, -0.01) | 0.0087 |

^1^CI is confidence interval; ^2^Model 1: run with smoking, alcohol consumption, physical activity, time spent watching TV, BMI category; Model 2: adjusted for sociodemographic variables: age, gender, income, education, marital status, living arrangements; and vitamin supplement use.

**Supplementary Table 2:** Unstandardised regression coefficients (95% CI) in a fully adjusted model according lifestyle factors among 2665 young adults (18-23 years) in Australia (2001-2004)^1 2^

| **Characteristics** | **Prudent pattern** |  | **Western pattern** |  |
| --- | --- | --- | --- | --- |
|  | **β (95% CI)** | ***P*-value** | **β (95% CI)** | ***P*-value** |
| Physical activity (sessions/wk) |  |  |  |  |
| Sufficient (≥5) | Reference |  | Reference |  |
| Insufficient (1-4) | -0.24 (-0.31, -0.18) | <.0001 | 0.28 (0.23, 0.34) | <.0001 |
| No physical activity (0) | -0.48 (-0.59, -0.37) | <.0001 | 0.37 (0.27, 0.47) | <.0001 |
| Time spent watching TV (h/d) |  |  |  |  |
| < 2 h | Reference |  | Reference |  |
| 2-4 h | -0.11 (-0.19, -0.04) | 0.0035 | 0.15 (0.08, 0.21) | <.0001 |
| ≥ 4 h | -0.26 (-0.35, -0.18) | <.0001 | 0.23 (0.16, 0.30) | <.0001 |
| Smoking (cigarettes/d) |  |  |  |  |
| Non-smokers | Reference |  | Reference |  |
| Smokers 1-19 | -0.12 (-0.19, -0.05) | 0.0007 | 0.12 (0.06, 0.18) | <.0001 |
| Smokers ≥20 | -0.40 (-0.55, -0.25) | <.0001 | 0.23 (0.10, 0.36) | 0.0004 |
| Alcohol consumption (standard drinks/d) |  |  |  |  |
| Non-consumers (0) | -0.07 (-0.20, 0.06) | 0.2881 | -0.01 (-0.13, 0.10) | 0.8442 |
| Light consumers (0.1-0.5) | Reference |  | Reference |  |
| Moderate consumers (0.51-1) | 0.07 (-0.02, 0.15) | 0.1235 | 0.02 (-0.05, 0.10) | 0.5259 |
| Heavy consumers (1.01-≥3.5) | -0.06 (-0.13, 0.01) | 0.1022 | 0.08 (0.02, 0.15) | 0.0092 |
| BMI (kg/m^2^) category |  |  |  |  |
| Normal (<25) | Reference |  | Reference |  |
| Overweight (25-30) | 0.09 (0.02, 0.17) | 0.0158 | -0.11 (-0.17, -0.04) | 0.0013 |
| Obese (≥30) | 0.12 (0.02, 0.21) | 0.0164 | -0.10 (-0.18, -0.02) | 0.0186 |

^1^CI is confidence interval; ^2^Models for each dietary pattern were run with the following lifestyle variables included; smoking, alcohol consumption, physical activity, time spent watching TV, BMI category. All results are adjusted for age in years, gender, income, education, marital status, living arrangement, vitamin supplement use and total energy intake.

**Supplementary Table 3**: Pearson’s correlations between dietary pattern scores and daily energy and energy adjusted intake of nutrients in 2665 young adults (18-23 years) in Australia (2001-2004)

| **Variables**^1, 2^ | **Western pattern** | ***P*-value** | **Prudent pattern** | ***P*-value** |
| --- | --- | --- | --- | --- |
| Energy, kcal | 0.76 | <0.0001 | 0.37 | <0.0001 |
| Fat, E% | 0.48 | <0.0001 | -0.40 | <0.0001 |
| Carbohydrate, E% | -0.48 | <0.0001 | 0.18 | <0.0001 |
| Protein, E% | -0.10 | <0.0001 | 0.17 | <0.0001 |
| Alcohol, %E | -0.09 | <0.0001 | -0.07 | 0.0001 |
| Protein, g | -0.07 | 0.0007 | 0.18 | <0.0001 |
| Carbohydrate, g | -0.26 | <0.0001 | 0.31 | <0.0001 |
| Total Fat, g | 0.31 | <0.0001 | -0.48 | <0.0001 |
| Saturated fat, g | 0.29 | <0.0001 | -0.48 | <0.0001 |
| Monounsaturated fat, g | 0.30 | <0.0001 | -0.39 | <0.0001 |
| Polyunsaturated fat, g | 0.03 | 0.1322 | -0.13 | <0.0001 |
| Cholesterol, g | 0.10 | <0.0001 | -0.07 | 0.0002 |
| Fibre, g | -0.37 | <0.0001 | 0.58 | <0.0001 |
| Sugars, g | -0.22 | <0.0001 | 0.25 | <0.0001 |
| Starch, g | -0.10 | <0.0001 | 0.11 | <0.0001 |
| Folate, µg | -0.33 | <0.0001 | 0.51 | <0.0001 |
| Vitamin C, mg | -0.18 | <0.0001 | 0.38 | <0.0001 |
| Vitamin E, mg | -0.12 | <0.0001 | 0.24 | <0.0001 |
| Retinol, µg | 0.12 | <0.0001 | -0.46 | <0.0001 |
| Retinol equivalent, µg | -0.11 | <0.0001 | 0.16 | <0.0001 |
| Riboflavin, mg | -0.22 | <0.0001 | 0.23 | <0.0001 |
| Niacin, mg | -0.20 | <0.0001 | -0.40 | <0.0001 |
| Niacin equivalent, mg | -0.18 | <0.0001 | 0.36 | <0.0001 |
| Thiamin, mg | -0.19 | <0.0001 | 0.21 | <0.0001 |
| Total β-Carotene, µg | -0.28 | <0.0001 | 0.52 | <0.0001 |
| Magnesium, mg | -0.41 | <0.0001 | 0.56 | <0.0001 |
| Calcium, mg | -0.24 | <0.0001 | 0.09 | <0.0001 |
| Iron, mg | -0.22 | <0.0001 | 0.35 | <0.0001 |
| Zinc, mg | -0.07 | 0.0006 | 0.17 | <0.0001 |
| Phosphorus, mg | -0.26 | <0.0001 | 0.33 | <0.0001 |
| Potassium, mg | -0.35 | <0.0001 | 0.53 | <0.0001 |
| Sodium, mg | 0.08 | <0.0001 | -0.11 | <0.0001 |

^1^Nutrient values are adjusted for total energy intake using residual method

^2^Nutrient values were log-transformed before adjusting for total energy intakes

**Supplementary Fig. 1**: Flow chart for selecting young adults to be included in the analysis at 21 year

Female participants who returned FFQ=1923

Male participants who returned FFQ=1731

3805 (52.7%) Total young adults at 21 year; 3654 returned FFQ (2001–2004)

76 Reported implausible energy

1847 Reported plausible energy intake

217 Had ≥40% missing items in FFQ

1630 Females included in the analysis

237 Reported implausible energy

1246 Males included in the analysis

1494 Reported plausible energy intake

248 Had ≥40% missing items in FFQ

1183 Males had useable dietary data to derive dietary patterns

63 Missing whole page of FFQ or other errors

1577 Females had useable dietary data to derive dietary patterns

53 Missing whole page of FFQ or other errors

7223 Total children at birth that established MUSP birth cohort (1981–83)

1135 Males had full data for all variables included in the analyses

1530 Females had full data for all variables included in the analyses
